# Protracted Course of Staphylococcus lugdunensis Septic Arthritis in Native Knee Joint

**DOI:** 10.7759/cureus.66848

**Published:** 2024-08-14

**Authors:** Sofia Howson, Sara L Ma, Jennifer Schmidt, Aakash Bisht, Teena Chopra

**Affiliations:** 1 Department of Internal Medicine, Wayne State University School of Medicine, Detroit, USA; 2 Department of Internal Medicine, Wayne State University Detroit Medical Center, Detroit, USA; 3 Department of Infectious Diseases, Wayne State University Detroit Medical Center, Detroit, USA

**Keywords:** staphylococcus lugdunensis, native joint, septic arthritis, social determinants, infectious diseases

## Abstract

*Staphylococcus lugdunensis* is a coagulase-negative bacteria of the Staphylococcus family. It is a highly invasive organism with similar virulence to *Staphylococcus aureus*. It is commonly associated with bacteremia and infections of the skin, soft tissues, joints, and bones. Those with indwelling medical devices are at the highest risk of infection due to biofilm formation. Instances of native joint infections are exceedingly rare. We describe a case of a 72-year-old female with multiple comorbidities presenting with native right knee joint septic arthritis from *S. lugdunensis*. Due to treatment noncompliance secondary to latent social determinants of health, she faced a complicated and protracted clinical course that was treated with inpatient intravenous antibiotics and outpatient oral doxycycline. Few cases of native joint infections with* S. lugdunensis* have been documented, and to our knowledge, the impact of treatment noncompliance on the sequelae of septic arthritis with this organism has not been reported. Socioeconomic factors and comorbidities have been shown to increase a patient’s risk for an extended joint infection with *S. lugdunensis.*

## Introduction

Staphylococcus lugdunensis (SL) is a coagulase-negative staphylococci (CoNS) found to be part of the normal skin flora [1]. In recent years, SL has been identified as an invasive disease with similar virulence to Staphylococcus aureus [1,2]. It has a higher pathogenicity than other CoNS and is mostly associated with infective endocarditis, bacteremia, bone and joint infections, and skin and soft tissue infections [1,3]. The typical source of CoNS infections is an indwelling medical device [4]. To this end, there have been many reports documenting the strong association of SL infection and prosthetic joint disease, with most cases occurring between six weeks and four years after implantation [1]. In contrast, patients with SL native joint infections are uncommon [5-7]. Our search of the literature uncovered only seven cases [5-11]. We report a case of a 72-year-old female with multiple comorbidities presenting with SL native knee joint septic arthritis, who had a prolonged clinical course due to an atypical septic joint presentation and treatment non-adherence secondary to uncommunicated social determinants of health. Our case report adds value to the existing literature by highlighting pertinent patient factors that need to be considered in treating this uncommon pathology.

## Case presentation

A 72-year-old female with a past medical history significant for end-stage renal disease (ESRD) on hemodialysis via permacath, heroin and cocaine use disorder, essential hypertension, seizure disorder, and previously treated hepatitis C infection presented to the emergency department with a chief complaint of right knee pain secondary to a mechanical fall one week ago. 

**Figure 1:**
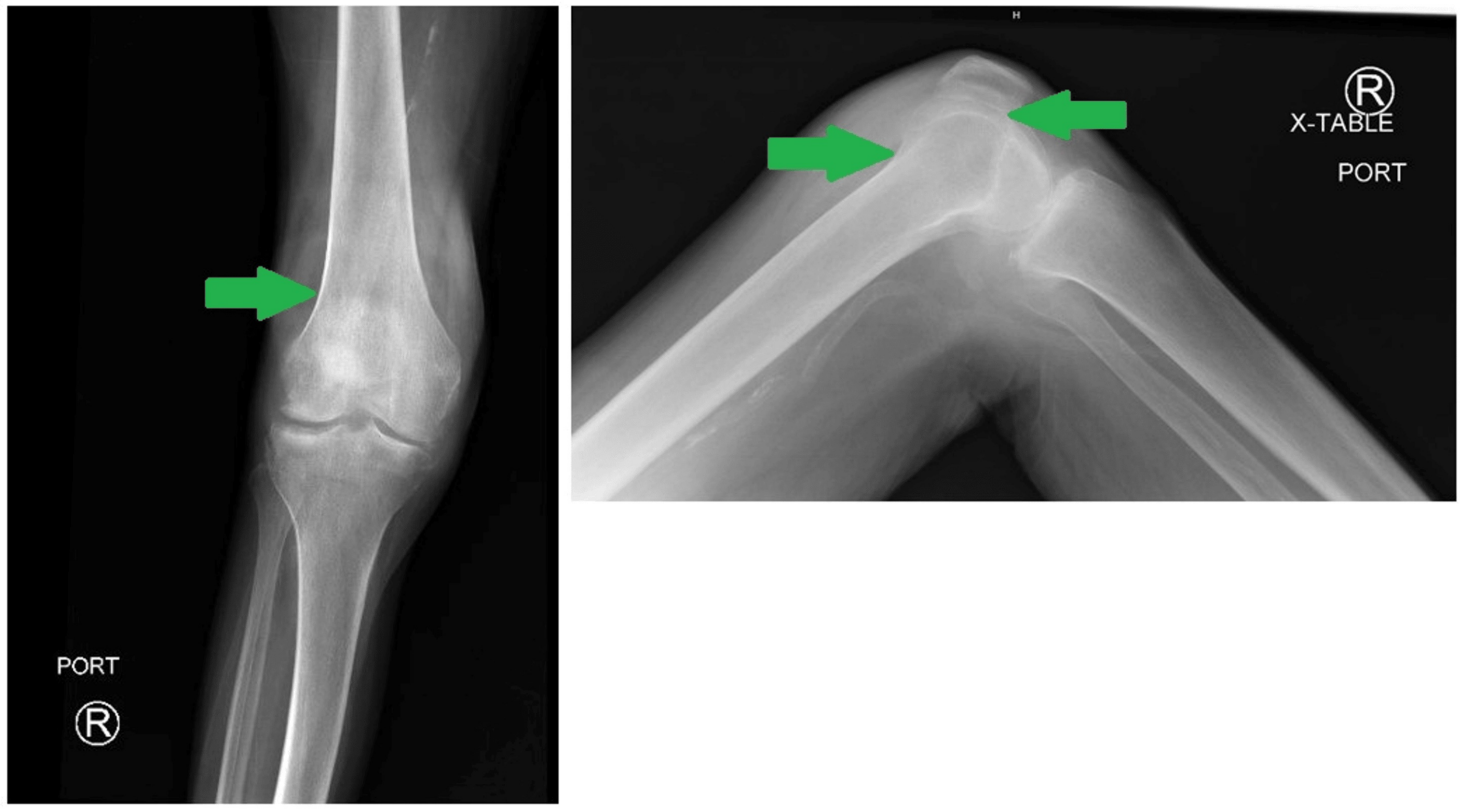
X-ray of right knee indicating joint effusion (green arrows)

**Figure 2:**
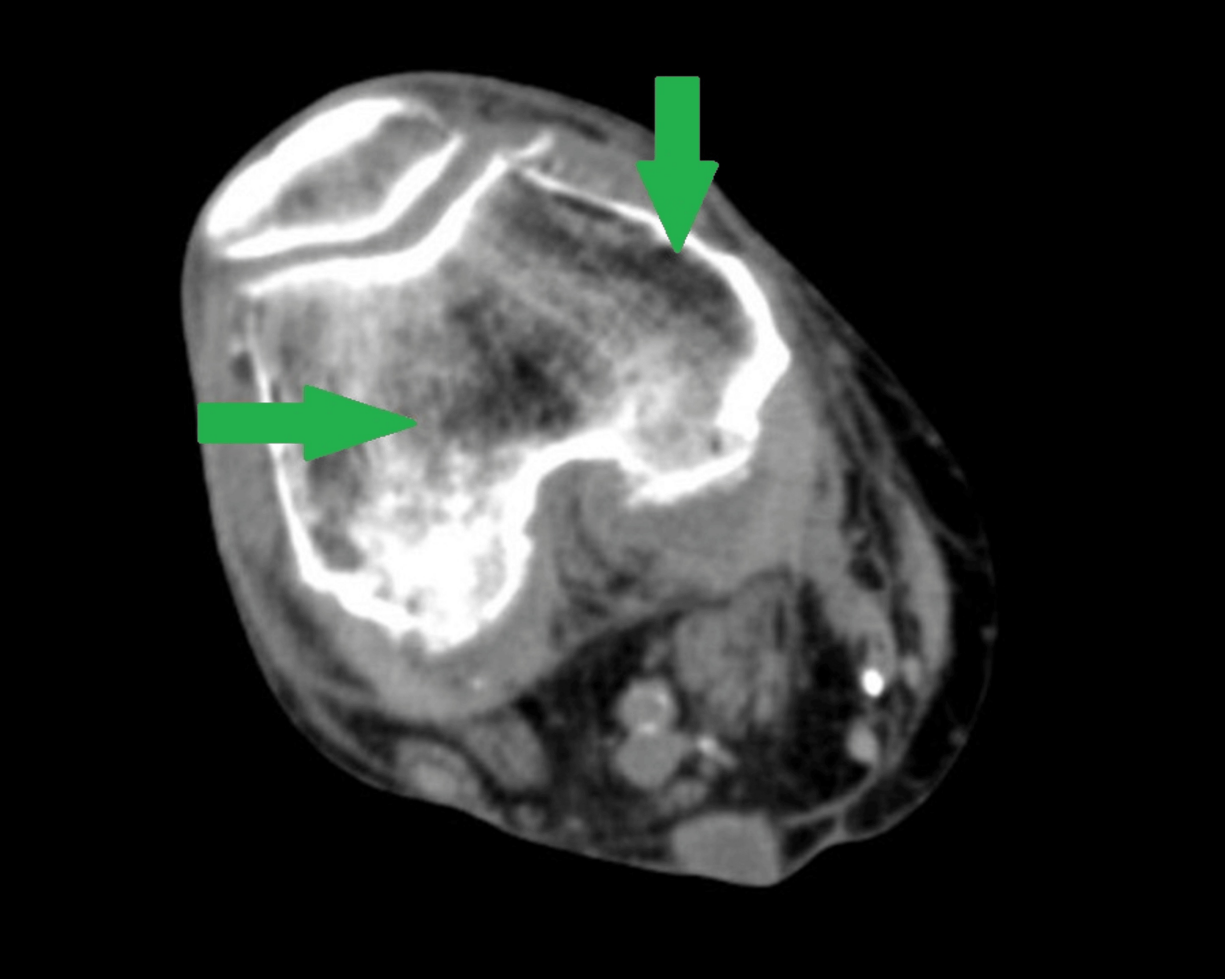
CT of right lower extremity showing joint effusion (green arrows) of the right knee

On the initial exam, the patient was afebrile with stable vital signs. Her right knee was swollen and tender to palpation with significantly reduced range of motion (ROM). The knee was not erythematous or warm. Her white blood cell count was within normal limits at 9,300 cells/mm3. X-ray (Figure 1) and CT (Figure 2) of the right knee showed joint effusions with advanced osteoarthritic changes. The patient was admitted for observation and workup. Orthopedics was consulted and joint aspiration was recommended. However, the patient refused and left the hospital against medical advice (AMA) on day four of hospitalization. At this point, the patient was only treated for opioid withdrawal. Due to the physical exam, relatively unremarkable imaging, and labs lacking a leukocytosis, the patient did not receive any antibiotics as the overall clinical picture of an afebrile, nonerythematous, and non-warm joint yielded low suspicion from the primary medicine team and orthopedics team for a septic joint. 

**Figure 3:**
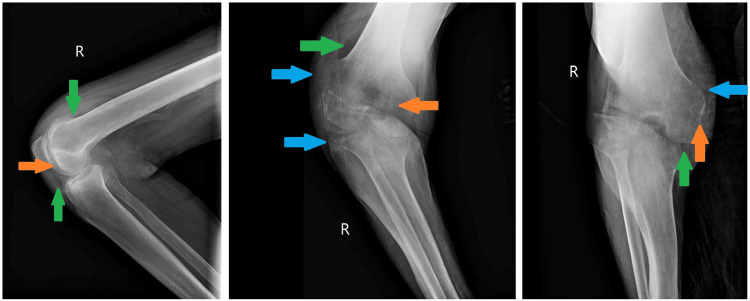
Three-view X-ray of right knee showing flexion deformity, mild degenerative changes (orange arrows), joint effusion (green arrows), and soft tissue swelling (blue arrows)

One day after leaving AMA, the patient returned to the hospital because she was unable to ambulate or perform any activities of daily living. She remained afebrile with normal vital signs, and a repeat X-ray of the right knee showed a flexion deformity with the same joint effusion (Figure 3). Laboratory analysis revealed leukocytosis, with a white blood cell count of 18,800 cells/mm3. The erythrocyte sedimentation rate (ESR) and C-reactive protein (CRP) levels were elevated at 115 mm/hour and 111 mg/L, respectively. A diagnostic arthrocentesis was performed, and joint fluid analysis revealed 177,000 nucleated cells with a neutrophilic predominance. The patient was started empirically on intravenous (IV) vancomycin and ceftriaxone. Blood cultures were taken throughout her hospitalization and repeatedly showed no growth. At 48 hours, cultures of the joint aspirate grew S. lugdunensis with resistance only to penicillin (Table 1). Antibiotics were consequently switched to IV cefazolin. The patient subsequently received two arthrotomies with irrigation and debridement. After 20 days of inpatient stay, the patient was comfortably ambulating with mild, well-controlled pain and was stable enough to be discharged home. She was transitioned from IV antibiotics to a 21-day outpatient course of oral 100 mg doxycycline taken twice daily and advised to follow up with her primary care provider within one week.

**Table 1:** Laboratory results depicting antibiotic susceptibilities of right knee joint culture <= Less than or equal to; S: Sensitive; R: Resistant.

Antimicrobial	Staphylococcus lugdunensis Minimum Inhibitory Concentration (MIC)	Interpretation
Clindamycin	<= 0.5	S
Erythromycin	<= 0.5	S
Gentamicin	<= 1	S
Oxacillin	0.5	S
Penicillin	0.5	R
Rifampin	<= 0.5	S
Tetracycline	<= 0.5	S
Vancomycin	1	S

Two days later, the patient returned to the emergency department with worsening right knee pain. Objectively, she was vitally stable and afebrile. Her labs, including a complete blood count, were within normal limits. She revealed that was not taking her antibiotics after being discharged due to lack of transportation to the pharmacy and insurance coverage. The patient was admitted, and during her stay, two more arthrotomies with irrigation and debridement were performed. The 21-day course of doxycycline was restarted, and after 16 additional days in the hospital, she was discharged to a skilled nursing facility to ensure completion of the antibiotic course. We assume complete resolution of symptoms, as our patient was lost to follow up and we have no further records of hospitalizations for this same complaint.

## Discussion

Our literature review revealed most cases of SL septic arthritis are associated with prosthetic joints. There are very few documented cases of SL septic arthritis of the native knee joint [5-11], with no previous documentation of prolonged symptom resolution due to treatment noncompliance. The majority of patients with either SL native or prosthetic joint infections also had a history of significant comorbidities or immunosuppression [1]. The most frequent comorbidities were pre-existing inflammatory joint pathology, diabetes mellitus, chronic steroid therapy, or some variant of a urogenital abnormality [1]. The patient presented in our case did have underlying high-risk health conditions including ESRD on hemodialysis via permacath and substance use disorder. These conditions may have predisposed her to a suboptimal immune response, making her more susceptible to a highly pathogenic SL infection.

Studies have found that infections from CoNS species are most commonly due to infection of an indwelling medical device [4]. This species creates a robust biofilm, which is one reason why it favorably infects prosthetic medical devices [12,13]. Although our patient did not have a prosthetic joint, she did have a left internal jugular vein permacath placed two years prior for her three times weekly dialysis treatments. Prior cases of S. lugdunensis bacteremia have been reported in patients receiving hemodialysis [12], and the permacath could be suspected to be a potential nidus of infection in our patient. However, this etiology is unlikely, as the catheter site was clean, dry, nonerythematous, nonpurulent, and non-tender, and regular blood cultures did not yield any growth. That is, our patient only had an isolated septic right knee joint without bacteremia.

Our case describes a protracted clinical course of SL native joint septic arthritis and underscores the role socioeconomic factors have in treatment adherence. S. lugdunensis has similar virulence factors to S. aureus, but it has much higher pathogenicity [1]. In studies comparing SL and S. aureus infective endocarditis, SL has been associated with mortality rates that are twice that of S. aureus [2,4]. Most often, this bacteria tends to be methicillin-sensitive with high susceptibility to a wide variety of antibacterial therapies [1,2,14]. Despite this, the ability of SL to form a biofilm makes it much more resistant to treatment, and is thus associated with a significantly higher complication rate and morbidity [1,13-15]. 

In our patient, the S. lugdunensis that was cultured from joint aspirate was susceptible to all antibacterials except penicillin. Although it was susceptible to doxycycline, a prolonged parenteral treatment was not favored due to lack of IV access in outpatient, as well as the concern that the patient may leave the hospital against medical advice, as she already had done so prior. Socioeconomic factors such as vehicle access and poverty are known to impact medication adherence, particularly with new prescriptions [16,17]. Socio-economic determinants can significantly affect the sequelae of S. lugdunensis septic arthritis particularly when the treatment plan involves daily outpatient medications and follow-up.

## Conclusions

Immunocompromised states and indwelling hardware are significant risk factors for native joint septic arthritis by S. lugdunensis. However, our case describes the possibility of native joint SL infection without orthopedic or other implantable devices providing a surface for biofilm formation. Although the patient did have an indwelling permacath and a history of IV drug use, multiple negative blood cultures and a clean site rule out the possibility of an infected device and bacteremia. She did report a mechanical fall one week prior, but did not present with any frank injuries. Since SL is part of natural skin flora, it is possible there was breakdown of the skin barrier at the time of injury that allowed for contiguous inoculation of the joint space. However, the lack of an obvious route of native joint space infection at the initial presentation is not surprising considering the organism’s high pathogenicity and virulence factors that function with unclear mechanisms. 

Socioeconomic factors do impact medication adherence, and we suspect that these factors also likely contributed to our patient’s observed sequelae of septic arthritis and loss to follow up. In conjunction with a clinical picture that was low-suspicion for septic arthritis, these factors can further lead to delayed diagnosis and interrupted antibiotic treatments. This exacerbates damage to the joint space over time and requires more complex and expensive medical intervention in the future. In our case, the patient’s protracted clinical course necessitated multiple irrigation and debridement procedures that could have been avoided with more appropriate interventions. Care teams should consider these socioeconomic factors prior to discharge to avoid complications and interruptions in treatment of highly virulent, persistent infections.
